# Neurocognitive deficits in a patient with small cell lung cancer: a case report

**DOI:** 10.1186/1757-1626-1-278

**Published:** 2008-10-27

**Authors:** Kanan H Hudhud, Ashiq Masood, Yun Oh, AZ Hegazi

**Affiliations:** 1 Cancer Care Center of Frederick, 46 B, Thomas Johnson Dr. Frederick, MD 21702, USA

## Abstract

**Introduction:**

The neurocognitive deficits associated with small cell lung cancer include metastasis to brain and paraneoplastic syndrome. The patients are also predisposed to herpes encephalitis due to immunosuppression and chemotherapy.

**Case presentation:**

We report a case of 72 year old female diagnosed with small cell lung cancer started with memory deficits, ataxia, vertigo, and hearing loss. The paraneoplastic work-up returned as anti-Hu antibody positive. The MRI brain showed area of edema and inflammation right medial temporal lobe as well as enhancement in the underlying meningi. Although PCR/HSV of CSF was negative, the patient was empirically treated with IV acyclovir and showed significant improvement and was discharged in good condition.

**Conclusion:**

The case highlights the importance of keeping broad minded approach in treating patients with serious malignant diseases. The case also call attention to the use of empiric therapy in possible life threatening diseases such as Herpes encephalitis

## Introduction

We report a case of 72 year old female diagnosed with small cell lung cancer started with neurocognitive deficits over the few weeks. The patient was empirically treated for suspected herpes encephalitis on the basis of clinical and MRI findings with acyclovir and showed significant improvement.

## Case presentation

**Figure 1:**
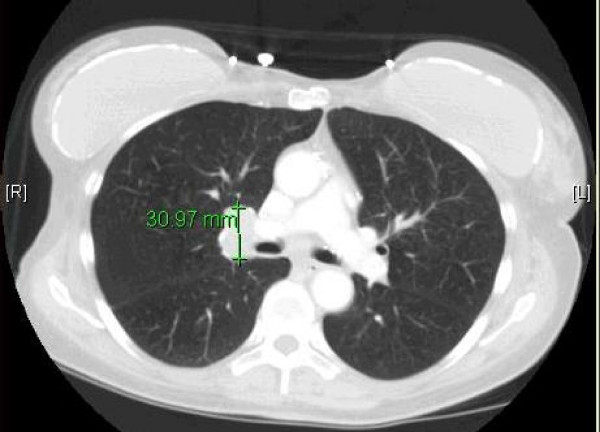
CT scan showing right hilar lymphadenopathy.

In May 2008, 72 year old female was diagnosed of small cell lung cancer incidentally after she was hospitalized for anxiety and dizziness. She was hyponatremic and series of tests which included a CT scan of the chest which showed right hilar lymphadenopathy and mediastinal lymphadenopathy (Figure 1). She then had a mediastinoscopy which was consistent with small cell lung cancer. PET/CT scan findings were consistent with CT scan. She was staged as limited stage small cell carcinoma of lung. She was started treatment with etopside and carboplatin. In July2008, after receiving two cycles of chemotherapy, she presented with new onset memory difficulties.

Further work-up included a MRI of brain with and without gadolinium as well as PET scan showed no evidence of metastatic disease to the brain. These symptoms were thought due to underlying anxiety or depression.

Few days later she started with ataxia, vertigo and hearing loss. She was prescribed meclizine which provided no relief. She then went to emergency department. At that time she was found to orthostatic. She was rehydrated and discharged.

Audiogram was done which revealed bilateral sensorineural hearing loss greater on the left side. The paraneoplastic workup was sent to rule out paraneoplastic syndrome. The anti-Hu antibody came positive. The diagnosis of anti-Hu syndrome was entertained. She was started on radiation therapy in addition to her chemotherapy for consolidation. She received 3960 cGy of radiation to the mediastinum in August 2008.

**Figure 2:**
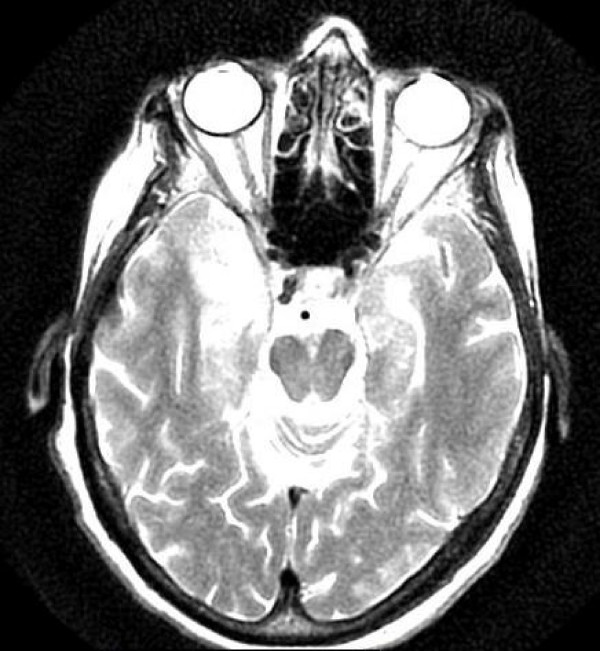
MRI showing abnormal signal intensity involving the medial aspect of the right temporal lobe.

Three weeks later, she began developing loss of smell sensation to the point that she was completely anosmic. Her unsteadiness reached to the point that she needed assistance for walking short distance. MRI of brain showed new area of edema and inflammation in the right medial temporal lobe as well as enhancement in the underlying meningi (Figure 2). She was admitted for to the hospital for empiric IV acyclovir therapy for possible herpes encephalitis. The Lumber puncture was done which showed increased proteins (Total proteins 5.7 g/l, Beta globulin of 0.73 g/l) and lymphocytes. The CSF HSV/PCR was negative. Patient was discharged in the 6 th day from the hospital and received total 10 days of IV acyclovir as she improved on the treatment. Patient is now planned for further chemoradiation therapy.

The neurocognitive deficits in our patient were probably because of either anti-Hu syndrome or Herpes encephalitis.

The anti HU syndrome is a neurologic paraneoplastic syndrome. A specific anti body called anti HU is present in serum and CSF of these patients [1]. Patients with anti-Hu antibodies usually have SCLC (94%), multifocal neurological symptoms and a poor prognosis [2]. Anti-Hu encephalomyelitis is one of the most frequent paraneoplastic syndromes, classically presenting with diffuse neurological involvement [3]. Anti-Hu antibodies are present in about 50% of patients with limbic encephalitis and lung cancer and is characterized by marked reduction in short term memory and sub acute confusion. Many patients may also develop rapid pancerebellar dysfunction due to extensive purkinjee neuronal loss termed as sub acute cerebellar ataxia. The HU-Abs is reported to be present in about 23% of these patients. The Anti-Hu antibodies, in addition to SCLC are associated with prostate cancer and neuroblastoma [4].

Anti-HU antibodies are also associated with Encephalomyelitis, sensory neuropathy, and autonomic neuropathy. The limbic encephalitis and sub acute cerebellar degeneration are associated relatively often with cancer and are called "classical" PNS [5]. The treatment of PNS is control of the tumor. Antitumor treatment has established to stop paraneoplastic neurological deterioration. However, in debilitated patients treatment of underlying tumor is withheld because of the little chance of clinically relevant neurological improvement [5].

Herpes simplex encephalitis patients may have a prodrome of malaise, fever, headache, and nausea that is followed by acute or sub acute onset of an encephalopathy whose symptoms include lethargy, Confusion, and delirium. Patients may also have seizures, aphasia, and other focal neurological deficits. The herpes encephalitis is fatal in 7 to 14 days if not treated and mortality as high as 70% in untreated cases. Neurology consultation should be sought as HSV encephalitis is neurologic emergency and treatment should be started with acyclovir in suspicious cases or when diagnosis is established. [6] In our case, the patient developed anosmia and that can be present in 65% of patients of HSV encephalitis [7]. However, PCR of spinal fluid for HSV was negative. PCR itself has a reported sensitivity of 98% and a specificity of 94% for the detection of HSV in the CSF [8].

False negative results may occur: [9]

1. Occasionally in bloody specimens through inhibition of PCR

2. When CSF has been obtained after starting Acyclovir.

3. When lumbar puncture has been performed early in disease.

4. When lumbar puncture was delayed approximately 10 days or more into the disease process

Since a negative HSV PCR result does not entirely rule out the possibility of HSE, an intrathecal antibody response specific to HSV should also be sought [9].

Although we did not prove the agent microbiologically but the MRI changes and Clinical improvement, elevated proteins and lymphocytes in CSF points towards HSV encephalitis. Our next step is try to get diagnosis retrospectively by measuring serial serum and CSF titers although it has no role in acute diagnosis and treatment [10].

## Conclusion

The case emphasizes the diagnostic challenge the patients with small cell lung cancer may present and clinicians may end up treating patients empirically for suspected infectious pathology. The case also demonstrates the need to use empiric therapy in suspected life threatening diseases such as Herpes encephalitis.

## Abbreviations

SCLC: Small cell lung cancer; HSV: Herpes simplex encephalitis.

## Consent

Written informed consent was obtained from the patient for publication of this case report and accompanying images. A copy of the written consent is available for review by the Editor-in-Chief of this journal.

## Competing interests

The authors declare that they have no competing interests.
